# Medicinal plants used by Tibetans in Shangri-la, Yunnan, China

**DOI:** 10.1186/1746-4269-5-15

**Published:** 2009-05-05

**Authors:** Yanchun Liu, Zhiling Dao, Chunyan Yang, Yitao Liu, Chunlin Long

**Affiliations:** 1 Kunming Institute of Botany, Chinese Academy of Sciences, Kunming 650204, PR China; 2 Graduate School, Chinese Academy of Sciences, Beijing 100039, PR China; 3 College of Life and Environment Sciences, China University for Nationalities, Beijing 100081, PR China

## Abstract

**Background:**

Medicinal plants used by the local people in Xizang (Tibet) have been investigated since the 1960s. The others out of Xizang, however, have been less understood, although they may be easily and strongly influenced by the various local herbal practices, diverse environments, local religious beliefs and different prevalent types of diseases. In 2006, two ethnobotanical surveys were organized in the county of Shangri-la, Yunnan Province, SW China, to document the traditional medicinal plants used by the Tibetan people.

**Methods:**

After literature surveying, four local townships were selected to carry out the field investigation. Three local healers were interviewed as key informants. The methods of ethnobotany, anthropology and participatory rural appraisal (PRA) were used in the field surveys. Plant taxonomic approach was adopted for voucher specimen identification.

**Results:**

Sixty-eight medicinal plant species in 64 genera of 40 families were recorded and collected. Among them, 23 species were found to have medicinal values that have not been recorded in any existing Tibetan literatures before, and 31 species were recorded to have traditional prescriptions. Moreover, the traditional preparations of each species and some folk medicinal knowledge were recorded and analyzed. These traditional prescriptions, preparations, new medicinal plants and folk medicinal knowledge and principles were discovered and summarized by local traditional Tibetan healers through times of treatment practices, and were passed down from generation to generation.

**Conclusion:**

As a part of the cultural diversity of Tibetan community, these traditional medicinal knowledge and experiences may provide data and information basis for the sustainable utilization and development of Tibetan medicine, and may contribute to the local economic development. However, for many reasons, they are disappearing gradually as time goes by. Our study showed that there were abundant traditional Tibetan medicinal prescriptions and using methods. It implies that more Tibetan medicinal plants and traditional knowledge can be discovered. Further research should be done to save the wealth of these traditional medicinal knowledge and experiences before they are dying out.

## Background

There are nearly 2000 ethnic groups in the world, and almost every group has its own traditional medicinal knowledge and experiences to depend on [1]. China is a multi-racial country with 56 nationalities, of which 55 in over 18 provinces are officially recognized as ethnic minorities. The various and abundant traditional ethnic medicines from each group have made up of the great Chinese medicine [2]. Tibetan medicine is one of the most important ethnic medicines with systematic theories, like the Han group’s medicine or the traditional Chinese medicine Chinese medicine which evolved out of the matrix of Chinese civilization over a period spanning more than four thousand years and developed into an extremely rich medical system [3-6]. Traditional Tibetan medicine which appeared in the seventh century, had later taken in the traditional Chinese medicinal theory, Indian medicinal theory, ancient Arabic medicinal theory, Greek medical theory and those of other countries around, and gradually became unique and well-known to the whole medicinal world [5,7-11].

During the long-term process of development of Tibetan medicine, many medical treatises had appeared, such as the *Yue Wang Yao Zhen *(Tibetan title: *Sman dpyad zla ba'i rgyal po*) [12], *Si Bu Yi Dian *(English title: the *Four Tantras*; Tibetan title: *Rgyud bzhi*) [13], *Si Bu Yi Dian Lan Liu Li *(English title: the *Blue Beryl Treatise*; Tibetan title: *Vaidurya Sngon Po*)[14] and *Jing Zhu Ben Cao *(Tibetan title: *Shel Gong Shel Phreng*) [15] which embodied the unique features of traditional Tibetan medicinal system with a relatively complete set of theory [5,16,17]. Actually, *Jing Zhu Ben Cao *is a widespread monograph mainly about medicinal herbals which described the morphological traits, the medicinal functions, the properties, the places of origin and the usage of Tibetan medicines. Comparing with *Jing Zhu Ben Cao*, other literatures are more emphasized on medical knowledge [1,5,10].

The Tibetan medicinal theory is based on the "Assumption of Five Elements" and the "Hypothesis of Three Aggregates". The "Assumption of Five Elements" believed that everything came from five elements of earth, water, fire, wind and space [5,10,18], so did the medicines. Moreover, it claimed that different combination of origins had formed six kinds of tastes of medicines, including sweet, sour, salty, bitter, pungent and astringent. In addition, eight traits and 17 curative effects of Tibetan medicine were concluded [5,10,18,19].

Recent studies on Tibetan medicine have shown that the earliest literature dates back to the eighth century AD [20]. Almost all the Tibetan inhabited areas, including Xizang (Tibet), Qinghai, Sichuan, Gansu and Yunnan provinces, have been investigated since the 1960s and the inventory of medicinal plants comprises 2600 species [8]. Furthermore, the phytochemical, pharmacological, new drug evaluation and other aspects of research on traditional Tibetan medicines have also attracted much attention and has become the hotspot in the medicinal field [1,5,7-11,16-34].

However, the medicines used by the branches of Tibetan communities distributed in other provinces may be easily and strongly influenced by the various local herbal practices, diverse environments, local religious beliefs and different prevalent types of diseases [16,34]. Consequently, local characters may have been formed in medicinal plants choosing, compositions of prescriptions and process of preparations which may be distinct from those in Tibet. Taking the Tibetan community in Yunnan as an example, the moving of their predecessors into northwest Yunnan dates back to the seventh century AD [35], and after living with other ethnic groups (the Yi, Naxi, Pumi communities and etc.) for such a long time, the Tibetans in Yunnan gradually formed its unique derivate style of using medicine due to various factors [20]. Though systematic theory had been formulated in the literatures mentioned above and the inventory of Tibetan medicines had also been studied, unfortunately few studies have been done on the derivate traditional knowledge and experiences of Tibetan medicines.

The local traditional Tibetan medicinal knowledge and managing experiences which are practiced, accumulated and passed down from generation to generation may play a significant role in the sustainable use and development of Tibetan plants resources [36,37]. Nevertheless, along with the disappearing of biodiversity and negative effects of mainstream culture, the traditional/folk medicinal knowledge of many ethnic groups is facing the danger of losing [38-41]. Furthermore, the losing of the traditional medicinal knowledge and culture which is the same as the disappearing of biodiversity is not a reversible process [2]. Therefore, it is imperative to carry out the systematic investigation and research on the local traditional Tibetan medicinal knowledge.

Diqing Tibetan Autonomous Prefecture, Northwest Yunnan, dominated by the Tibetans, is one of the spreading areas of traditional Tibetan medicine. Because of its topographic diversity, unique location and climate condition, a great number of plants, animals and minerals are used as medicine [42]. Anderson and his group had been fascinated by the Tibetan medicines in the Mt. Kawa Karpo in Diqing Tibetan Autonomous Prefecture and a vegetation analysis was made there [34], but no effort on recording the traditional Tibetan medicinal knowledge was made. In another case, aiming at making the scientific name of the medicines clear, Prof. Yang Jingsheng had been to Xizang (Tibet) and Northwest Yunnan many times since 1964 to investigate the Tibetan medicines, and had written a book "Tibetan Medicines in Diqing Tibetan Autonomous Prefecture" containing more than 679 species of Tibetan medicines. Furthermore, the revision or confirmation of the medicinal uses, the language derivation of Tibetan dialect and the name of 40 species of Tibetan medicinal plants were included [2]; some known chemical compositions and pharmacological achievement of Tibetan medicines were also simply recorded [43]. However, he also did not carry out any ethnobotanical studies on the local traditional Tibetan medicinal knowledge and experiences.

For these reasons, we conducted an ethnobotanical study containing the local traditional prescriptions, preparations, uses and other local characters on Tibetan medicinal plants in Shangri-la, and hoping that the local traditional medicinal knowledge and experiences would be recognized, saved and enriched, and the data and information of the study result would contribute to the sustainable use and development of Tibetan medicine through the study.

### Study area

**Figure 1:**
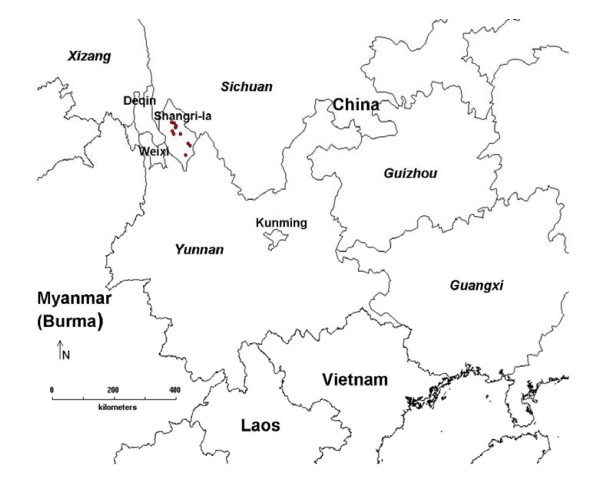
**Study area – Shangri-la County**.

Shangri-la County (Figure 1), formerly named as Zhongdian County, located between 26°52' 28°48' N and 99°23' 100°31'E, is one of the three counties administered by Diqing Tibetan Autonomous Prefecture, Northwest Yunnan. It is located in the south of Qinghai-Tibet Plateau and the east of Himalayas, and the junction of Yunnan, Tibet and Sichuan provinces. The world-famous area called Three Parallel Rivers (Nujiang River or Salween River, Lancang River or the Mekong River, and Jinsha River or Upper Yangtze River) covers the whole Shangri-la County whose total area accounts 11,613 km^2^. Shangri-la is the largest county in Yunnan Province but has a lowest population density of 10.46 people/km^2 ^with a population of nearly 130,000. Besides the dominant nationality of Tibetan in Shangri-la, there are also Han, Naxi, Yi, Bai communities, among which mutual cultural influence and communications have existed for a long time [44].

The terrain of Shangri-la is higher in north and lower in south. The place with the highest altitude of 5,545 m above sea level in Shangri-la is the Gezong Snow Mountain in North, and the one with the lowest altitude of 1,503 m is the Laohuofang Village in Luoji Township in south. Between the two altitudes, the climate zone of Shangri-la is divided into North Semi-tropical Zone, Temperate Warm Zone, Temperate zone, Temperate Cold zone, Frigid Zone and Glacier Zone. Abundant medicinal resources are distributed in Shangri-la under its unique geological location and climate condition [44].

The ethobotanical study on local traditional medicinal knowledge and experiences in Shangri-la may represent the derivate branch of Tibetan medicinal knowledge of Diqing Tibetan Autonomous Prefecture, NW Yunnan.

## Materials and methods

According to the usual tools and methods used in conducting ethnobotanical study, we carried out our work in following 4 steps: literature surveying, sites selection, field study and herbarium work. Participatory Rural Appraisal (PRA) [45-50], ethnobotanical and anthropological methods was applied during the process of investigation and field study [44,51,52].

### Literature surveying

Relevant literature were surveyed and consulted to obtain the information on the topography of Shangri-la and information on the Tibetan medicine and traditional healers which were helpful in choosing the specific study sites [1,29,42-44]. The general Tibetan medical information was also obtained by reading books and literatures before field study according to the guide manual on the ethnobotanical methods [48,51].

### Selecting sites and searching local healers

We selected four local townships of three different altitudes at which the vegetation type distributing is different to carry out our investigation: Xiao Zhongdian Township with an altitude of 3,240 m, Nixi Township of 3,160 m, Luoji town of 2,196 m and Jinjiang Township of 1,900 m [44].

Guiding by the principles of direct learning from local people of PRA [45-49], we widely asked local Tibetans about the local healers in their villages and towns. During the searching and talking, we found that most Tibetans in Yunnan, especially the official workers, traders and local healers who have to contact with different people very often, now can speak basic mandarin. So we all speak mandarin and didn't use any interpreters in communicating.

Finally, we found Qiao Ruxiang, a local healer from Xiao Zhongdian Township, Gu Jiangzhao, a herbalist from Jinjiang Township and Wang Za, a local healer from Nixi Township. Among them, Wang Za is the only one who had attended the Chinese Primary School built all over China but not the Tibetan Buddhism School used to open only for Lama in Shangri-la. They all knew some Tibetan medicinal theory which passed down from their ancestors, and they knew few about the famous Tibetan medical treatises in history.

### Field study

The field study was carried out twice in July and August, 2006, in addition to several pilot investigations in the region.

Before the field study, we discussed with the local healer, asked about his opinion and made decisions together on where to go and what to do in the following few days. Our intention to enable the local healers to conduct their own analysis, and to plan and take action is accord with one of the main principles of PRA [45-50] and achieved good result in the end.

During the field study, some of us collected specimens of medicinal plants known by the healers and conversations naturally came about the medicinal plants, the related knowledge and experiences. The healers were the main talker, and we gave suitable questions and responses according to what they said. One of us was responsible for writing down all the answers and information given by the healers. The open-ended interview of anthropological technique was applied here [48].

We didn't found any local herbalist in Luoji town, but fortunately we widely collected some plants there, of which some were later recognized by Wangza from Nixi Township as medicinal plants used by Tibetans.

Under the guide of local traditional Tibetan healers, specimens of medicinal plants which are commonly used by the healers were collected, and the local Tibetan pronunciations, traditional prescriptions, preparations and medicinal usages of the plants were recorded according to the requirement of making inventory of ethnobotanical methods [51,52]. Concerning about the local Tibetan pronunciations, we would like to state here that they are claimed different from the formal Tibetan pronunciation in Tibet by local Tibetans. Some of the plants were even pronounced like Chinese. We didn't change the name and just wrote them down phonetically.

As the traditional Tibetan healers are so few that we could hardly find more, or in some cases, some healers were too old to guide us in the field. Therefore it seems even more urgent to collect the traditional Tibetan medicinal knowledge and experiences from folk healers before the traditional knowledge and experience dying out.

### Studies in herbarium (KUN)

Plants specimens were examined and identified by experts and the authors. The specimens were registered and were under further study before being deposited in the Herbarium of the Kunming Institute of Botany, Chinese Academy of Sciences (KUN).

## Results and Discussions

### Sixty-eight Tibetan Medicinal Plants

Through this study, 68 medicinal plant species belonging to 64 genera in 40 families were collected. All the 68 species are listed in alphabetical order in Additional file 1, Appendix S1. Twenty-three of the 68 medicinal plant species were found to have medicinal values that have not been recorded in any literatures related to medicinal plants in China and neighborhood countries before [16,43,53-55], and no records of them were found in a online databases of a resource and information centre for 7300 edible and other useful plants [56] (labeled "*" in appendices attached to this paper). Moreover, the database gives a clue of a lot of useful literatures on the medicinal uses of plants [57-87] which are important for studying medicinal plants. The voucher specimen number, botanical family, local Tibetan pronunciations, plant parts/product used, the traditional preparations and uses of all the 68 plants are also recorded and reported in the present paper.

The main altitude range of medicinal plants distributed in Tibet is from 3,600~5,200 m [88]. Nevertheless, due to the geological restrictions, 30.9% of the medicinal plants collected under the guide of local Tibetan healers were from 3,000~3600 m in Shangri-la, and 69.1% were from below the altitude of 3,000 m (27.9% from 2800~3000 m, 32.4% from 2100~2700 m, and 8.8% from 1900~2000 m). This altitude range directly influences the medicinal plants species being used.

This number of traditional Tibetan medicinal plants represents only a small fraction of the flora of Shangri-la. Among the 40 families, Asteraceae is the one with the most medicinal plants in our study (seven species), followed Labiatae and Umbelliferae (5 species each). Fifty-seven (83.8%) of the medicinal species are Dicotyledons, seven are Monocotyledons (10.3%), two are ferns (2.9%), one is fungus (1.5%) and one is gymnosperm (1.5%). The percentages of the plant groups in this study are close to that in "Tibetan Medicine in Diqing Tibetan Autonomous Prefecture" with 86.6% of Dicotyledons, 8.3% of Monocotyledons, 1.2% of fungi, 2.9% of ferns and 0.3% of gymnosperm (Table 1).

**Table 1:** The percentage of main plant groups

	**The Percentage of Species from the Medicinal Plants in our Study (%)**	**The Percentage of species in "Tibetan medicine in Diqing Tibetan Autonomous Prefecture" (%)**
**Fungi**	1.5	1.2
**Lichens**	0	0.6
**Algae**	0	0.1
**Pteridophyta**	2.9	2.9
**Gymnospermae**	1.5	0.3
**Dicotyledoneae**	83.8	86.6
**Monocotyledoneae**	10.3	8.3
**Total**	100	100

### Thirty-one medicinal plants with 33 prescriptions

Among the 68 medicinal plant species, 31 have been written down with 33 traditional prescriptions. Most ingredients of the prescriptions are plants, of which the local name and the botanical Latin name were written down; some ingredients are things like liquid wax, tallow and muskiness. All the plants have been listed in alphabetical order with the prescriptions (Additional file 1, Appendix S2).

Though there are only 33 local traditional prescriptions of 31 plants, these prescriptions are recorded neither in "Tibetan Medicine in Diqing Tibetan Autonomous Prefecture" [42] nor in the famous ancient Tibetan medicinal monograph of "Jing Zhu Ben Cao" mentioned above [15]. The prescriptions' compositions of one species are relatively simpler than that of the species of the same genus in Tibet which are both easily attainable in Shangri-la and Tibet respectively [89]. Few prescriptions are even derivate from other ethnic groups in Yunnan, such as that of the "*Phytolacca acinosa *Roxb." which was learned to from Yi community [90].

### Inventory of plant parts/product used

The plant parts/product used for medicinal purposes are shown in the table (Table 2), and the roots (27%) may be concluded as the most frequently used part, and the leaves (16%) follows the second. However, the stem (6%) is the second frequently used one if we count the rhizome (11%), tuber (6%) and bulb (1%) in, i.e. 23% in total. Local traditional healers consider all the underground part as roots, including the rhizome, tuber, bulb and underground sclerotium. In this philosophy, the underground part which accounts 47% was more frequently used than the overground one in local Tibetan medicine.

**Table 2:** Plant parts/product used

**Plant Part/Product**	**Number of uses**	
		%
Roots	22	27
Leaves	13	16
whole plant	11	14
Rhizome	9	11
Tuber	5	6
Stem	5	6
Fruits	4	5
Seeds	3	4
Bark	3	4
Bulb	1	1
Twig	1	1
Turpentine	1	1
underground sclerotium	1	1
Fruits	1	1
Flowers	1	1

**Total**	81	100

		%
Underground	38	47
Overground	32	39
whole plant	11	14

**Total**	81	100

The classic concepts on the plant part used claim that the overground part and whole plant accounts a large percent of 70% [84] in traditional Tibetan medicine which are more than that (53%) of this study. We ascribe the reasons to the environment the local healers dwelled in and the medicinal plants the healers usually collected.

### Types of preparations and main diseases

According to the "*Jing Zhu Ben Cao*" (Tibetan title: *Shel Gong Shel Phreng*) and other modern literatures [15,88,91], there are six types of preparations: powders, pills, adhesive plasters, decoctions, medicinal wines and medicinal butters. Similar to the traditional preparations documented in "*Jing Zhu Ben Cao*", decoction (63%) and powder (18%) are commonly used, but medicated wine (4%) is not so often used (Table 3). However, distinct from the traditional preparation types, plants being stewed with meat or steamed with egg which is thought to be easily assimilated and have favorite tastes are also recommended by local healers.

**Table 3:** Preparation methods

**Kind of Preparation**	**Number of Species with Different Preparation**	**Occupied Percentages (%)**
Decoction	43	63
Powder	12	18
Crushing fresh plant	4	6
Stewing with meat	3	4
Medicinal wine	3	4
Steaming with egg	2	3
Turpentine	1	1

**Total**	68	100

The 68 traditional Tibetan medicinal plants may cure 37 kinds of diseases, among which the dysentery is the most common illness treated (Table 4). Comparing with the 10 common efficacies of traditional medicines in Tibet (cure cold/relieving a fever, clearing away heat from visceral organs, curing tracheitis, controlling tuberculosis, relieving rheumatism, antalgic uses, reducing the blood pressure, curing fractures and gynaecological diseases) [88], only four diseases among the first ten prevalent ones in this study are as the same (cold/fever, fractures, rheumatism and gynaecological diseases), which implies the prevalent diseases in Shangri-la are different from those in Tibet.

**Table 4:** Diseases/symptoms cured by of the 68 plants

	**Number of diseases**	
		%
Dysentery	11	8.73
Gastropathy	10	7.94
Cold, fever	10	7.94
Rheumatism arthritis	9	7.14
Fractures	7	5.56
Cough	6	4.76
Sciatica	6	4.76
Wounds	6	4.76
Toxic condition	6	4.76
Gynaecological diseases	6	4.76
Promoting circulation of blood	4	3.17
Diuresis	4	3.17
Feebleness	4	3.17
Sterility	3	2.38
Toothache	3	2.38
Tracheitis	2	1.59
Internal hemorrhage	2	1.59
External hemorrhage	2	1.59
Inflammation	2	1.59
Hepatitis	2	1.59
Dyspepsia	2	1.59
Neuralgia	2	1.59
Enteritis	2	1.59
Contusions	2	1.59
Hemoptysis	1	0.79
Pneumonia	1	0.79
Measles	1	0.79
Hemorrhoids	1	0.79
Ophthalmic disease	1	0.79
Cardialgia,	1	0.79
Food poisoning	1	0.79
Ascites, hydropsy	1	0.79
Cystitis	1	0.79
Anthelmintic	1	0.79
Calvities	1	0.79
Chilblain	1	0.79
Antenatal pain	1	0.79

**Total**	126	100

### Four tastes of medicines

Interesting principles are expressed by one (Wang Za from Nixi township) of the three traditional Tibetan healers in Shangri-la. Different from the six tastes of Tibetan medicine in "Jing Zhu Ben Cao", four tastes are concluded by them including bitter, pungent, sour and fishy. Furthermore, plants with a bitter taste are thought to be helpful in controlling inflammation and relieving pain; pungent plants may contribute to control inflammation and cure a cold; sour ones are considered to be useful in warming the internal organs, especially the stomach; plants with a fishy tastes may relive a cough and clear away heat in lung.

### Choosing medicines by altitude

Moreover, instead of plants from higher altitude, the same plant species colleted in place with lower altitude are only taken as medicine by person from higher altitude, and people inhabited in place with higher altitude would like to take medicinal plants from lower altitude. The local Tibetan healers explained that the plants from different altitude may contain some substances which may be lacked of in the plants from the same altitude, and taking the plants from different altitude as medicine would be more helpful in curing diseases.

These principles and knowledge are explained by authors to be close related to the living environment of Tibetan in Shangri-la. The terrain of Shangri-la is higher in north and lower in south and Tibetan are distribute both in south and north, so did medicinal plants. For example, the plants of *Saussurea *sp. are actually have better curative effects if being collected above an altitude of 3,000 m and snow line; and "*Phytolacca acinosa *Roxb." grows better in place of lower altitude and will be more helpful when being used as medicine.

## Conclusion

The main altitude range of the study sites in Shangri-la are between 1,900~3,600 which directly influences the medicinal plant used there. Including the local prescriptions' compositions, the dominant plant part being used, the types of preparations, the common diseases cured, the local four tastes of medicine and choosing medicines by altitude, all the traditional medicinal knowledge and principles are distinct with local characters from that in Tibet. This traditional knowledge which was accumulated by local healers through making use of the special plant resources in Shangri-la and learning from treatment practices and others' experiences has its own character and local style due to the important impacts from local geological environments, climate and cultural conditions, herbal practices and different prevalent types of afflictions in Shangri-la.

Furthermore, the traditional medicinal knowledge with local characters which was summarized by local traditional healers was accumulated and passed down from generation to generation. The investigation and documentation of these local traditional principles, experiences and knowledge will not only be an inheritance and spreading of traditional Tibetan medicinal knowledge but also be a supplement and conservation of the wealth of Tibetan cultural diversity and culture legacy. And these traditional medicinal knowledge and experiences may also play a significant role in conserving local biodiversity and contribute to the local economic development [92].

Moreover, the 23 plant species which are newly showed to have medicinal values imply that there are much more local Tibetan medicinal plants and traditional knowledge waiting to be discovered and recorded. Both medicinal plant species newly discovered and the folk medicinal knowledge and culture recorded will provide basic data and information to the sustainable utilization and development of Tibetan medicine, to the new drug development and to the further research of traditional patent medicines [89,93].

However, during the period of investigating, most traditional Tibetan healers were found to be almost 70 years old, and their descendants are scarcely willing to inherit this traditional profession and the precious traditional knowledge handed down They prefer other jobs with more income instead. They believe that the traditional medicines are not so indispensable to their life nowadays and their reasons are given as follows: 1) the medical treatment of Han community is more advanced and has better condition than the traditional ones; 2) Tibetan became more and more dependent on the money-consuming substances outside their village, but traditional healers didn't earn much.

It can be concluded that because of the long-term influence from Han community's culture and way of life, traditional Tibetan medicinal culture are facing the danger of dying out which would be a great loss not only to the Tibetan cultural wealth but also to the great culture diversity of China. It is urgent that we should carry out further study on the traditional medicinal knowledge and experiences handed down in the folk to prevent its disappearing and make the best of it.

It is possible to give appropriate suggestions on how to promote the sustainable utilization of Tibetan medicinal resources and the local economic development only when the Tibetan medicinal resources had been fully investigated and the further ethnobotanical study on local traditional Tibetan medicinal knowledge and experiences had been done.

## Competing interests

The authors declare that they have no competing interests.

## Authors' contributions

Author YCL performed the interviews with the healers, identified the herbarium specimens with CYY, drafted and finalized the manuscript with CLL. Author ZLD joined YCL to perform interviews. Author CYY joined YCL to perform interviews, identified herbarium specimens with YCL. Author YTL joined YCL to perform interviews. Author CLL supervised the research works and finalized the manuscript with YCL.

## Supplementary Material

Additional file 1 **Appendices**. Appendix 1 Medicinal plants used by the Tibetans in Shangri-la and Appendix 2 Thirty-three traditional prescriptions of 31 medicinal plant species. Click here for file
