# Supplementary material for: Medicinal plants used by Tibetans in Shangri-la, Yunnan, China
Source: J Ethnobiol Ethnomed. 2009 May 5;5:15. doi: 10.1186/1746-4269-5-15 (PMC2684741; doi:10.1186/1746-4269-5-15)
Supplement: Additional file 1 — Appendices. Appendix 1 Medicinal plants used by the Tibetans in Shangri-la and Appendix 2 Thirty-three traditional prescriptions of 31 medicinal plant species. [file 1746-4269-5-15-S1.pdf]

Appendix 1. Medicinal plants used by the Tibetans in Shangri-la

Scientific Name (Voucher Specimen Number and Family Name)	Local Pronunciations	Part(s)/Product Used	Ethnomedicinal Preparations and Uses
Abutilon paniculatum Hand.-Mazz. (1645, Malvaceae) *	zha ma	Roots & leaves	Roots and leaves are consumed to moisten the intestine.
Acanthocalyx alba (Hand.-Mazz.) M. Cannon (1528, Dipsacaceae)	\	Roots	Decoction is prepared to cure gynaecological diseases including emmeniopathy.
Acanthocalyx nepalensis (D.Don) M. Cannon (1440, Dipsacaceae) *	\	Roots	Roots are stewed with meat and are eaten as tonic.
Agrimonia pilosa var. *nepalensis* (D. Don) Nakai (1584, Rosaceae) *	xian he cao	Roots	Decoction is prepared to cure dysentery and enteritis.
Ajuga forrestii Diels (1624, Labiatae)	ku dan cao	Rhizome	Decoction is consumed to cure dysentery.
Anaphalis busua (Buch.-Ham. ex. D. Don) DC. (1603, Asteraceae)	hie gen wo lü	Leaves	Powder of leaves is applied on the wound caused by cutting and shooting to heal it. The plant is also considered to have veterinary effects.
Anemone hupehensis Lem. (1642, Ranunculaceae)	ye mian hua	Rhizome	Decoction is used as anthelmintic.
Anemone rivularis Buch.-Ham. ex DC. (1607, Ranunculaceae)	hu zhang cao	Rhizome	Decoction is prepared to cure bronchitis. Powder is consumed with water to cure the same diseases. The plant is considered to have veterinary uses.
Arctium lappa Linn. (1575, Asteraceae)	xue rong mu	Fruits, leaves & roots	Decoction has medicinal uses as detoxification, relieving fever, controlling internal hemorrhage and curing measles, dysentery and gastropathy.

Arisaema erubescens (Wall.) Schott (1517, Araceae)	yi dua	Tuber	Slices of tuber are dipped into brine for 2 days, then cleaned, dried and ground, then put the powder into a fresh clean gallbladder of cattle, expose the gallbladder to sunlight and do not change the gallbladder until the former one is dried. The powder can not be used until it has been put into 9 gallbladders and waited for at least one year later (the longer the better). It is to relieve a cough, cure hemoptysis and control pneumonia.
Arisaema saxatile Buchet (1587, Araceae) *	dua	Tuber	Decoction is prepared to relieve a cough and control the chronic bronchitis. It is also considered to have veterinary uses by traditional healers.
Asparagus myriacanthus Wang et S. C. Chen (1652, Liliaceae)	tian men dong	Tuber	Tuber is considered to have tonic and hematinic uses.
Astilbe rivularis Bich.-Ham. ex D. Don (1562 & 1601, Saxifragaceae) *	lü sheng ma	Roots	Crushed roots are mixed with wine and applied on body parts to set a broken bone.
Bidens pilosa L. (1619, Asteraceae)	yi bao zhen	Leaves	Leaves are steaming with egg and are eaten to cure hemorrhoids and anal hemorrhage.
Boenninghausenia albiflora (Hook.) Reichenb. ex Meisn (1650, Rutaceae)	yang bu chi cao	Whole plant	Whole plant is used to cure a cold.
Bupleurum petiolulatum Franchet (1516, Umbelliferae) *	chai hu	Whole plant	Decoction is prepared to cure a cold.
Cannabis sativa L. (1627, Cannabiaceae)	suo luo	Stem & leaves	Decoction has medicinal uses as emmenagogue and invigorating the circulation of blood.
Clematis rehderiana Craib (1634, Ranunculaceae)	ba zhong	Stem & leaves	Decoction is prepared to wash hair to cure calvities.
Cornus capitata Wall. (1621, Cornaceae)	yi mu	Fruits, stem and leaves	Fruits, twig and leaves are used as veterinary medicine. Fruits are also eaten as food.
Cuscuta japonica Choisy	yi che ke ta	Vine	Decoction is prepared to cure

(1633, Convolvulaceae)			hepatitis.
Cynanchum otophyllum Schneid. (1647, Asclepidaceae) *	ba be da	Roots	Roots are stewed with meat and are eaten to cure rheumatism.
Cynoglossum amabile Stapf et Drumm. (1611, Boraginaceae)	ci jia mie diu	Whole plant	Decoction is to cure dysentery. And the plant with white flowers is considered to have better medicinal uses.
Cypripedium flavum P. F. Hunt et Summerh. (1401, Orchidaceae)	zang a mie diu	Rhizome	Rhizome is used as detoxification medicine, especially when having food poisoning.
Datura stramonium L. (1626, Solanaceae)	\	seeds & flowers	Crushed seeds are applied on the teeth to relieve toothache. Flower is steeping in wine with a proportion of 1/1000, and a small quantity of wine are drank to cure rheumatism.
Drosera peltata Smith (1529 & 1665, Droseraceae)	yong na zong nuo	Bulbs	Bulbs are stewed with chicken and are eaten to cure women's eye itching and conjunctivitis. Nine fresh bulbs are swallowed to cure cardialgia.
Drynaria sinica Diels (1628 & 1719, Drynariaceae)	gu sui bu	Rhizome	Powder is applied on body parts to set a broken bone and diminish inflammation; Decoction is prepared to stimulate the circulation of the blood and cause the muscles and joints to relax.
Galium glandulosum Hand.-Mazz. (1639, Rubiaceae) *	qiang zuo	Roots	Decoction of roots and other ingredients is prepared to cure sciatica and rheumatic neuralgia.
Geranium sinensis R.Knuth (1612, Geraniaceae) *	ge shan xiao	Roots	Decoction is consumed to help digesting and cure dysentery and gastropathy. The plant is also considered as veterinary medicine.
Gerbera nivea Sch.-Bip. (1622, Asteraceae) *	bai tou weng	Whole plant	Decoction is prepared to cure dysentery.
Gnaphalium hypoleucum DC. (1593, Asteraceae)	xie xie hie gen wo lü	Leaves	Powder is applied to heal cutting and shooting wounds. It is considered to have veterinary effects.

Grifola umbellata Pilat (1549, Polyporaceae) *	pa jia tuo ti	underground sclerotium	Decoction is prepared to promote diuresis and to cure ascites and hydropsy.
Habenaria davidii Franch. (1615, Orchidaceae) *	liang xiong di	Tuber	Decoction is prepared to cure sterility.
Halenia elliptica D. Don (1613 & 1618, Gentianaceae)	qing ye dan	Whole plant	Decoction of whole plant parts is consumed to cure hepatitis and dysentery. It is also considered as veterinary medicine.
Hedera nepalensis var. *sinensis* (Tobl.) Rehd. (1643, Araliaceae)	mer lü、a guo lü guo	Stem & leaves	Decoction is prepared to set a broken bone and cure sciatica.
Hypericum bellum Li (1637, Hypericaceae)	tu lian qiao	Fruits & leaves	Decoction helps preventing catching a cold, and decoction of leaves is prepared to cure roup.
Leontopodium sinense Hemsl. (1592, Asteraceae) *	hie gen wo lü	Leaves	Powder is applied to heal cutting and shooting wounds. The plant is also considered to have veterinary effects.
Leonurus japonicus Houttuyn (1648, Labiatae)	suo ye、suo pu	Whole plant	Whole plant is used to cure emmeniopathy.
Ligusticum brachylobum Franchet (1614, Umbelliferae)	be xie mu	Roots	Decoction is consumed to relieve a cough and cure a cold.
Maianthemum fusciduliflorum (Kawano) S. C. Chen & Kawano (1600, Liliaceae) *	wo bong	Rhizome	Rhizome is used to promote diuresis.
Meeboldia yunnanensis (H. Wolff) Constance & F. T. Pu (1636, Umbelliferae) *	\	Whole plant	Decoction of the whole plant is used to relieve a cough and cure a cold.
Morus alba L. (1646, Moraceae)	\	Roots, bark and twig	Decoction is prepared to relieve a cough and cure neuralgia.
Origanum vulgare L. (1574, Labiatae)	le ren ne ba	Whole plant	Whole plant is cut up and stir-fried, and then boiled. Decoction is to cure a cold and clean the bowel by purging when having food poisoning.
Paeonia delavayi var. *lutea* (Delavay ex Franch.) Finet et Gagnep. (1556,	a duo luo duo	Bark	Decoction is to cure rheumatism and gastropathy.

Paeoniaceae)			
Panax japonicus (Nees)C. A. Meyer (1480 & 1522, Araliaceae)	ge da qi	Rhizome	Rhizome is stewed with meat and eaten as tonic; Powder of rhizome is applied to set a broken bone and stanch a cut.
Phytolacca acinosa Roxb. (1590, Phytolaccaceae)	tuo qiong	Roots	Roots are crushed and applied on the navel to promote diuresis.
Pimpinella candolleana Wight & Arn (1545, Umbelliferae) *	yang shan chou	Roots	Roots are cut up and steamed with egg, and the mixture is eaten to cure gastropathy.
Pinus densata Mast. (1559, Pinaceae)	no peng	Turpentine	Turpentine to cure arthritis and arthrosis gall.
Pleurospermum rivulorum (Diels) K. T. Fu & Y. C. Ho (1602, Umbelliferae)	qiang huo	Roots	Decoction is used to clear away heat, cure dysentery, stimulate the circulation of the blood, cause the muscles and joints to relax and prevent catching a cold. Powder is applied to set a broken bone and diminish inflammation.
Polygonum macrophyllum D. Don (1609, Polygonaceae)	lang qu	Rhizome	Powder is consumed with water to cure gastropathy and dysentery.
Polygonum paleaceum var. *pubifolium* Sam. (1598, Polygonaceae) *	rang qu	Rhizome	Powder is consumed with water to cure gastropathy and dysentery.
Potentilla anserina L. (1591, Rosaceae)	fan bai ye	Roots	Decoction is consumed to control leukorrhea flow. It is also considered to have veterinary uses.
Prunus mira Koehne (1635, Rosaceae)	ka muo	Nutlets	Relieving a cough and cure injuries from falls, fractures, contusions and strains.
Psammosilene tunicoides W. C. Wu et C. Y. Wu (1608, Caryophyllaceae)	du ding zi	Roots	Roots getting rid of bark are dried and ground, and powder is taken with water to cure gastropathy or is applied to stanch a cut.
Pteris cretica L. (1640, Pteridaceae) *	feng wei cao	Whole plant	Decoction is prepared to promote diuresis and cure cystitis.
Rumex nepalensis Spreng. (1548, Polygonaceae)	a gong bu li	Roots	Decoction of roots is prepared to help digesting and purging and to cure gastric diseases and stomach swelling.

Salvia flava Forrest ex Diels (1519, Labiatae)	da hong shen	Roots	Decoction is prepared to cure emmeniopathy and stimulate the circulation of the blood.
Salvia trijuga Diels (1583, Labiatae)	xiao hong shen、yong zhu mu	Roots	Decoction is prepared to cure diseases such as having much menstrual flow and sterility. The plant is also considered as veterinary medicine.
Satyrium yunnanense Rolfe (1361, Orchidaceae) *	liang xiong di	Tuber	Decoction is prepared to cure sterility.
Senecio laetus Edgew. (1625, Asteraceae) *	tu san qi	Whole plant	Decoction is prepared to cure rheumatism.
Sinopodophyllum hexandrum (Royle) T.S Ying (1594, Berberidaceae)	a ga mie le	Seeds	Decoction is to cure antenatal pain happened before or after childbirth and help expelling placenta.
Sophora davidii (Fr.) Komarov ex Pavol (1632, Papilionaceae)	cha ke mu	Seeds	Crushed seeds are applied on teeth to control toothache.
Stellera chamaejasme f. *chrysantha* S. C. Huang (1525, Thymelaeaceae)	shu she mie diu	Roots	Powder of roots is consumed with water to cure stomach swelling and help purging to clean the bowel when having food poisoning. Roots are dried in sun without meeting rain, or they would turn green and became poisonous.
Thalictrum javanicum Bl. (1539, Ranunculaceae)	jin si huang lian	Roots	Decoction of roots will be consumed to cure dysentery and enteritis, to prevent catching a cold, to allay a fever and help detoxification. The plant is also considered to have veterinary uses.
Verbascum thapsus L. (1571, Scrophulariaceae)	hie shong	Leaves	Fresh leaves are roasted to yellow and crushed, applying leaves on body parts to heal a cut.
Verbena officinalis L. (1623 & 1672, Verbenaceae)	\	Roots	Decoction of roots is consumed to control toothache and cure dysentery.
Viburnum kansuense Batal. (1446, Caprifoiaceae) *	\	Fruits	Fruits are steeped into wine and used as tonic.

Viola tienschiensis W.Becker (1630, Violaceae) *	pen ba chong chong	whole plant	Decoction is applied on body parts to cure chilblain and heal cut.
Wikstroemia ligustrina Rehd. (1586, Thymelaeaceae) *	lue ran me	Bark	Decoction is prepared to set a broken bone and to cure sciatica and injuries from falls, fractures, contusions and strains.

Appendix 2. Plant-based traditional prescriptions used by the Tibetans in Shangri-la

Scientific Name	Prescriptions
Acanthocalyx alba (Hand.-Mazz.) M. Cannon	Roots are boiled with "tu yang shen" (*Triplostegia glandulifera* Wall.), "bai ling shen" (plants belong to Araliaceae), "yi mu cao" (*Leonurus heterophyllus* Sweet), "xiao hong shen" (*Salvia trijuga* Diels) and "da hong shen" (*Salvia flava* Forrest ex Diels).
Agrimonia pilosa var. *nepalensis* (D.Don) Nakai *	Roots are boiled with "rang qu" (*Polygonum paleaceum* var. *pubifolium* Sam.), "ge shan xiao" (*Geranium sinensis* R. Knuth) and "chuan xin lian" [*Andrographis paniculata* (Burm. f.) Nees].
Ajuga forrestii Diels	Rhizome is boiled with "bai tou weng" (*Gerbera nivea* Sch.-Bip.) in water.
Anemone hupehensis Lem.	Rhizome is boiled with the bark of "*Punica granatum* L.", the seeds of "*Melia azedarach* L.", the tube of "*Stemona japonia* (BL) Miq." and the seeds of "*Cucurbita moschata* Duch".
Anemone rivularis Buch.-Ham. ex DC.	Rhizome is boiled with "ban xia" [*Pinellia ternata* (Thunb.) Breit.] and another unknown ingredient.
Arctium lappa Linn.	Fruit, leaves and roots are boiled with "rang qu" (*Polygonum paleaceum* var. *pubifolium* Sam.) and "ge shan xiao" (*Geranium sinensis* R. Knuth) in water.
Arisaema saxatile Buchet *	Tubers are boiled with “ren shen” (*Panax ginseng* C. A. Mey.) and “gan cao” (*Glycyrrhiza uralensis* Fisch.) in water.
Cannabis sativa L.	Stem and leaves are boiled with "xiao hong shen" (*Salvia trijuga* Diels) and "da hong shen" (*Salvia flava* Forrest ex Diels).
Cuscuta japonica Choisy	Bine and leaves are boiled with "qing ye dan" (*Halenia elliptica* D. Don) and "ku dan cao" (*Ajuga forrestii* Diels) in water.
Datura stramonium L.	Crushed seed are mixed with liquid wax.
Geranium sinensis R. Knuth *	Roots are boiled with "rang qu" (*Polygonum paleaceum* var. *pubifolium* Sam.) and "ge shan xiao" (*Geranium sinensis* R. Knuth) in water.
Gerbera nivea Sch.-Bip. *	Whole plant parts are boiled with "qing ye dan" (*Halenia elliptica* D. Don).
Grifola umbellata (Pers.:Fr.) Pilat *	Sclerotium is boiled with "xiao mu tong" (*Clematis armandii*

	Franch.), "fu ling" [*Poria cocos* (Schw.) Wolf], "shi wei" [*Pyrrosia lingua* (Thunb.) Farwell], "wu gong cao" (*Pteris vittata* L.), "ma bian cao" (*Verbena* officinalis L.), "bian xu" (*Polygonum aviculare* L.) and "che qian cao" (*Plantago asiatica* L.).
Habenaria davidii Franch. *	Tubers are boiled with "tu yang shen" (*Triplostegia glandulifera* Wall.), "bai ling shen" (plants belong to Araliaceae), "xiao hong shen" (*Salvia trijuga* Diels) and "*Acanthocalyx nepalensis* (D.Don) M. Cannon" in water.
Ligusticum brachylobum Franchet	Roots are boiled with "*Aster tataricus* L. f." in water.
Paeonia delavayi var. *lutea* (Delavay ex Franch.) Finet et Gagnep.	Dried bark is boiled with "*Euonymus vagans* Wall." and "*Magnolia officinalis* var. *biloba* Rehd. et Wils.".
Phytolacca acinosa Roxb.	Roots are crushed with muskiness.
Pinus densata Mast.	Turpentine is applied with tallow.
Pleurospermum rivulorum (Diels) K. T. Fu & Y. C. Ho	Roots are boiled with the bark of plants of "*Berberis*", the leaves of plants of "*Eucalyptus*" species, "wu gong cao" (*Pteris vittata* L.), "fang feng" [*Saposhnikovia divaricata* (Turcz.) Schischk.] and "huang qin" (*Scutellaria baicalensis* Georgi) in water.
Polygonum macrophyllum D.Don	Rhizome is boiled with "ge shan xiao" (*Geranium sinensis* R. Knuth) or fried rhizome is ground with "ge shan xiao" .
Polygonum paleaceum var. *pubifolium* Sam. *	Rhizome is boiled with "ge shan xiao" (*Geranium sinensis* R. Knuth) or fried rhizome is ground with "ge shan xiao".
Potentilla anserina L.	Roots are boiled with "xiao hong shen" (*Salvia trijuga* Diels) and "da hong shen" (*Salvia flava* Forrest ex Diels).
Prunus mira Koehne	Nutlets and almond are used together.
Pteris cretica L. *	The whole plant is boiled with "xiao mu tong" (*Clematis armandii* Franch.), "che qian cao" (*Plantago asiatica* L.) and "fu ling" [*Poria cocos* (Schw.) Wolf].
Salvia flava Forrest ex Diels	Roots are boiled with "xiao hong shen" (*Salvia trijuga* Diels), "da hong shen" (*Salvia flava* Forrest ex Diels) and "fan bai ye" (*Potentilla anserine* L.) in water.
Salvia trijuga Diels	①: Roots are boiled with "da hong shen" (*Salvia flava* Forrest ex Diels) and "fan bai ye" (*Potentilla anserina* L.) . ②:Roots are boiled with "liang xiong di" (*Habenaria davidii* Franch./ *Satyrium yunnanense* Rolfe), "tu yang shen" (*Triplostegia glandulifera* Wall.), "bai ling shen" (plants belong to Araliaceae) and "*Acanthocalyx nepalensis* (D.Don) M. Cannon".
Satyrium yunnanense Rolfe *	Tubers are boiled with "tu yang shen" (*Triplostegia glandulifera* Wall.), "bai ling shen" (plants belong to Araliaceae), "xiao hong shen" (*Salvia trijuga* Diels) and "*Acanthocalyx nepalensis* (D.Don) M. Cannon".
Stellera chamaejasme f. *chrysantha* S. C. Huang	Roots are ground with "tu da huang" (*Rumex nepalensis* Spreng.), "chen xiang" [*Aquilaria sinensis* (Lour.) Gilg] and "ba dou" (*Croton tiglium* L.).

Thalictrum javanicum Bl.		①: Boiling the dried roots with "rang qu" (*Polygonum paleaceum* var.*pubifolium* Sam.), "xian he cao" [*Agrimonia pilosa* var. *nepalensis* (D.Don) Nakai], "bai tou weng" (*Gerbera nivea* Sch.-Bip.), "ci jia mie diu" (*Cynoglossum amabile* Stapf et Drumm.) and "Berberis" species. ②: Dried roots are boiled with "wu gong cao" (*Pteris vittata* L.), dried tangerine peel, "jin yin hua" (*Lonicera japonica* Thunb.), the bark of "*Phellodendron amurense* Rupr." and plant of "*Berberis*" species.
Viola tienschiensis W. Becker	*	Whole plant is boiled with "tu da huang" (*Rumex nepalensis* Spreng.).
Wikstroemia ligustrina Rehd.	*	Bark is boiled with "*Acer buergerianum* Miq.", the roots of "qiang zuo" (*Galium glandulosum* Hand.-Mazz.) and the twig of "*Morus alba* L.".
